# Review of successful pathways for regulatory approvals in open-field fluorescence-guided surgery

**DOI:** 10.1117/1.JBO.26.3.030901

**Published:** 2021-03-13

**Authors:** Brian W. Pogue, Eben L. Rosenthal

**Affiliations:** a Thayer School of Engineering at Dartmouth, Center for Imaging Medicine, Hanover, New Hampshire, United States; b Norris Cotton Cancer Center, Dartmouth-Hitchcock Medical Center, Lebanon, New Hampshire, United States; c Stanford University School of Medicine, Palo Alto, California, United States

**Keywords:** surgical, fluorescein, contrast, indocyanine green, cancer, perfusion

## Abstract

**Significance:** The modern use of fluorescence in surgery came iteratively through new devices and pre-existing imaging agents, with indications that were paved via regulatory approvals and device clearances. These events led to a growing set of surgery subspecialty uses.

**Aim:** This article outlines the key milestones that initiated commercially marketed systems and agents by highlighting temporal sequences and strategic decisions between them, with the goal of helping to inform future successes.

**Approach:** A review of successful regulatory approvals and the sequences between them was completed for companies that achieved US Food and Drug Administration (FDA) premarket approval or new drug approvals (NDAs) or device clearances in the fields of fluorescent imaging agents, open surgery imaging devices, and their approved medical indications.

**Results:** Angiography agents, indocyanine green and fluorescein, were approved for human use as absorbing dyes, and this use in retinal imaging was the precursor to lateral translation into tissue perfusion imaging in the last two decades with a growing number of devices. Many FDA cleared devices for open fluorescence-guided surgery used the predicate created by the SPY SP2000 system. This first system was 510(k) cleared for angiography imaging with a unique split predicate from x-ray imaging of vasculature and retinal fluorescence angiography. Since that time, the lateral spread of open surgery devices being cleared for new indications has been occurring with a growth of adoption in surgical subspecialties. Growth into new surgical subspecialties has been achieved by leveraging different NDAs and clearances between indications, such that medical uses have broadened over time.

**Conclusions:** Key decisions made by developers to advance specific device clearances and NDAs have been based upon existing optical fluorescent agents. The historical lessons and regulatory trends in newer indications and contrast agents can help the field evolve via successful investment in new systems and applications.

## 1 Introduction

To have a significant impact in clinical use and the subsequent patient outcomes, fluorescence-guided surgery (FGS) requires US Food and Drug Administration (FDA) approved commercial drugs and devices for each specific medical indication. The lessons learned from approval of one imaging agent or imaging system, can inform the regulatory pathway for a completely different surgical specialty or indication. Dissemination of these imaging agents and devices has been ubiquitous in the field of FGS and it is this diffusion of ideas that has helped the field progress.^1^ A study of the FDA approvals and clearance processes in the past can help us to see trends and key milestones that are shaping what is possible clinically. Company investment in imaging agents and devices drives the field forward, and result in the regulatory applications and approvals found on the FDA website. While many regulatory review papers are forward-looking perspectives,^2^ few have examined the history of approvals with an eye to learning from past lessons. In this article, we complete a historical review of key milestones and pathways for approved devices/agents with a focus on: (i) fluorescent imaging agents; (ii) fluorescent imaging devices for surgery; and (iii) charting the sequence of indications where they were approved for use, and the linear connections between them.

**Fig. 1:**
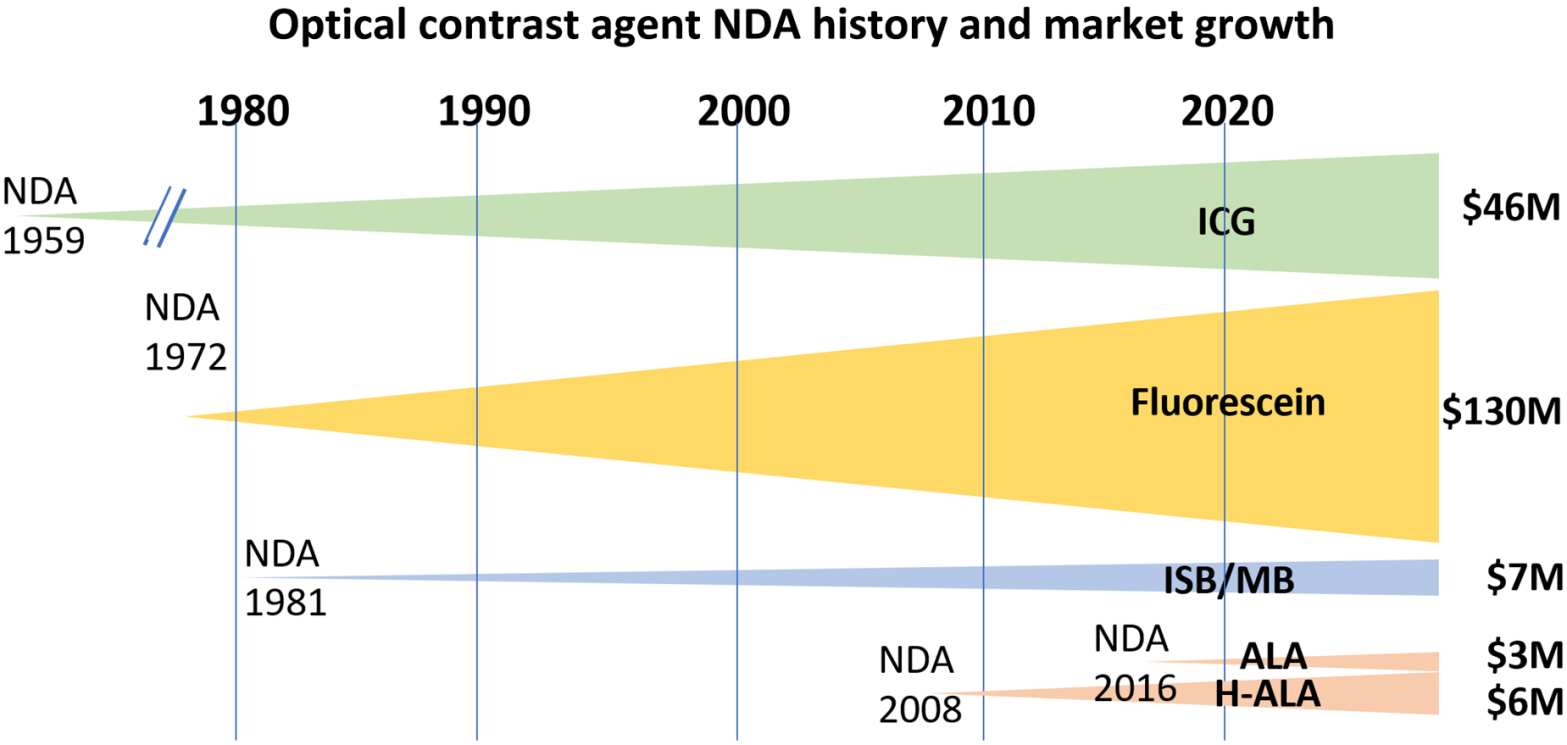
Optical imaging agent development has its origins dating back to the early to middle of last century, with ICG, fluorescein, and MB each having been used in multiple human studies prior to their NDA dates, and each originally approved for other applications, such as an absorbing agent in the cases of ICG and fluorescein and as a therapeutic in the cases of MB and ALA. The modern use of ALA as a pre-cursor to fluorescence from heme metabolism started after its use in the 1990s as a photodynamic agent, and subsequent realization of its fluorescence imaging potential in the early 2000s.

The main driver in the field of FGS is the fluorescent imaging agents used, and so analysis of the field should start with these. New imaging agent “new drug approvals” (NDAs) are approved by the FDA, and surprisingly all agents used for fluorescence imaging today were originally approved not as fluorophores, but for their other features. The earliest approval was indocyanine green (ICG) in 1959 as a green pigmented dye for use as a visible imaging agent. Fluorescein followed this in 1972, similarly as a visible-light absorbing imaging agent used in conjunction with a densitometer to measure hepatic function and cardiac output testing.^3^^–^^6^ Figure 1 shows four major optical agents and their financial impact in the field. While ICG has only become highly successfully used in fluorescence imaging within in the last 15 years, the growth in the use of fluorescein has been steady in retinal imaging for decades. These two vascular flow agents dominant the FGS market. A related footnote is the research ongoing with fluorescence from methylene blue (MB) and isosulfan blue;^7^^–^^10^ although they are not approved as fluorescent agents, they are indicated for use as blue dyes for visible contrast to guide breast surgery^11^^–^^14^ and MB as a treatment for methemoglobinemia.^15^^,^^16^ The most advanced tissue-specific fluorophore has been forms of aminolevulinic acid (ALA), which is metabolically converted to fluorescent protoporphyrin IX (PpIX) in the mitochondria of active cells and was originally approved as a topical photodynamic therapy agent for skin lesions in 1999 as Levulan^®^.^17^^–^^19^ ALA is generally a cellular metabolism-specific agent because PpIX is produced by the heme synthesis pathway, instead of vascular or perfusion specific, and so it has unique imaging characteristics that affect the imaging system design, mostly in terms of sensitivity and wavelength band. ALA in various forms has seen approvals in bladder cancer detection^20^^,^^21^ and neurosurgical glioma image guidance.^22^^,^^23^ Strategies for approval of investigational agents are closely held industry trade secrets. However, a careful review of the publicly available documents for approved agents may help us understand the history of approaches to approval for new agents in medical indications.

Although fluorescent agents are key to generating sufficient signal to background, the device platform is critical for successful in situ imaging. Commitment to an imaging device for identifying anatomy or for oncological clinical trials must consider aspects of the tissue type, surgical approach, cost, and the fluorophore properties. The pivotal step in pre-marketing clearance for such an indication is through filing an application with the FDA. While it might be commonly thought that these devices are approved with the use of an existing NDA imaging agent, it is interesting to recognize that market approval/clearance of systems as an imaging agent-device combination was initially more common, but that this is decreasing in relative numbers as the field broadens. The “combination product” route at the FDA is complex because of the need to have staff review both the new imaging agent and the new device.^24^ Recent changes in regulatory statutes and policy in the FDA Reauthorization Act of 2017, have also led to fewer combination product designations by the Office of Combination Products. Additionally, from the industry perspective, the complexity of good manufacturing practices for both the imaging agent and the device is high,^25^ and quite different in nature, so the staff involved both at the company and the FDA increases and the bar for approval of both is necessarily high. So, more commonly now companies are seeking to approve or clear either an imaging agent or a device, one at a time. This reduces financial risk and makes FDA decision-making simpler and perhaps more transparent, but also allows the company to focus its products in one core area instead of two.

The relative risk of the device and intended use results in the device categorization of Class I, II, or III. Most devices are designated Class II, and so clearances are achieved through the 510(K) pathway, where a new device is proven by the company to be safe and effective by being substantially equivalent to an existing cleared device.^26^ This approach, considered the path of least resistance, and cost, become an argument in justification and logic in the application to try to show that two devices are substantially similar in their intended use, even if they have very significant differences in other ways. For Class III devices, a Premarket Approval (PMA) application is required for devices that “…support or sustain human life, are of substantial importance in preventing impairment of human health, or which present a potential, unreasonable risk of illness or injury.” However, there are few of these applications in the FGS field, mostly because the indications being sought are as a visualization tool in surgery. Most manufacturers developing devices for indications such as tissue perfusion have been successful in gaining clearance for their devices as Class II technology.

Historically, FDA has required device manufacturers to submit PMAs for indications that involve claims of cancer detection. In the assessment of these devices, it is critical to remember how the imaging information will be used during the procedure by the surgeon, and that this forms just one piece of information, whereas the surgeon will consider all the information available to them at the time including visual and tactile information, aided also by frozen section pathology. As with other aspects of optical imaging technology regulation, the FDA may be further refining its position on this classification of FGS systems. One example of such regulatory evolution is the De Novo application. Prior to the De Novo option, a device with a Class II risk was automatically re-designated as a Class III device requiring an PMA submission, if no substantially equivalent predicate device exists and the 510(k) application fails.^27^ The De Novo path offers another route by which a new device can demonstrate its indication safely and appropriately without the burden of a Class III designation. In all these premarket filings with the FDA, the device can be cleared for sale if the application is deemed successful, but the strategy of which pathway to choose occurs early on and can be extremely expensive if chosen incorrectly, or highly cost saving if an easier path is found. These costs directly contribute to the success or failure of the field, and so a study of these regulatory issues is synergistic with the development and testing of device technology.

An examination of trends in clearances for the same indication can be illuminating. Alternatively, additions of indications to an existing approved device are a well-established pathway to broaden its intended use and subsequent market. Specifically, there are important lessons in the synergy across different surgical sub-specialties. The device clearances that have occurred across technologies have been critically important to advance FGS, for example where an indication in vascular flow in retinal imaging can be used to justify an indication in tissue flap surgery to assess vascular perfusion. There have not been that many explicit examples of crossing sub-specialty, as often companies focus within a subspecialty and work on a range of technologies within that field; however, these cross-specialty indications are key to expanded use of FGS.

Recently, several 510(k) clearance applications from different companies have clustered around a set of indications in open surgery, where devices are cleared by a single predicate. This pathway in a proven medical indication leads to the fastest commercial success, although it can be limiting for the field if there is insufficient penetration to yield success from multiple devices. In the end, comparisons of clearance pathways that have been successful in translation will be important, given that this interdisciplinary cross communication is often driven both by the companies and between surgeons and specialties. This review takes a historical view of the pathways and describes the commercial successes in that context.

## 2 History of New Imaging Agent Approvals as Fluorescent Agents

The history of retinal angiography is longer than any other use of exogenous fluorescence in medicine^3^^,^^5^^,^^28^^,^^29^ with experimental use dating back to 1961, and FDA NDA occurring in 1972. Fluorescence angiography of the eye evolved early and is dominated by fluorescein because of its value in surface imaging angiography with the Heidelberg retinal angiograph. However, imaging of deeper choroidal circulation was not possible with this, and so ICG was used as an imaging agent for this lower layer of the eye.^30^^,^^31^ The absorption mode was used initially, and later fluorescence was exploited.^29^^,^^32^^,^^33^ Still despite the differences in use, the market for fluorescein in fundus imaging appears stronger than ICG (see Fig. 1), indicating that the value of surface imaging appears stronger than subsurface, in this indication.

**Fig. 2:**
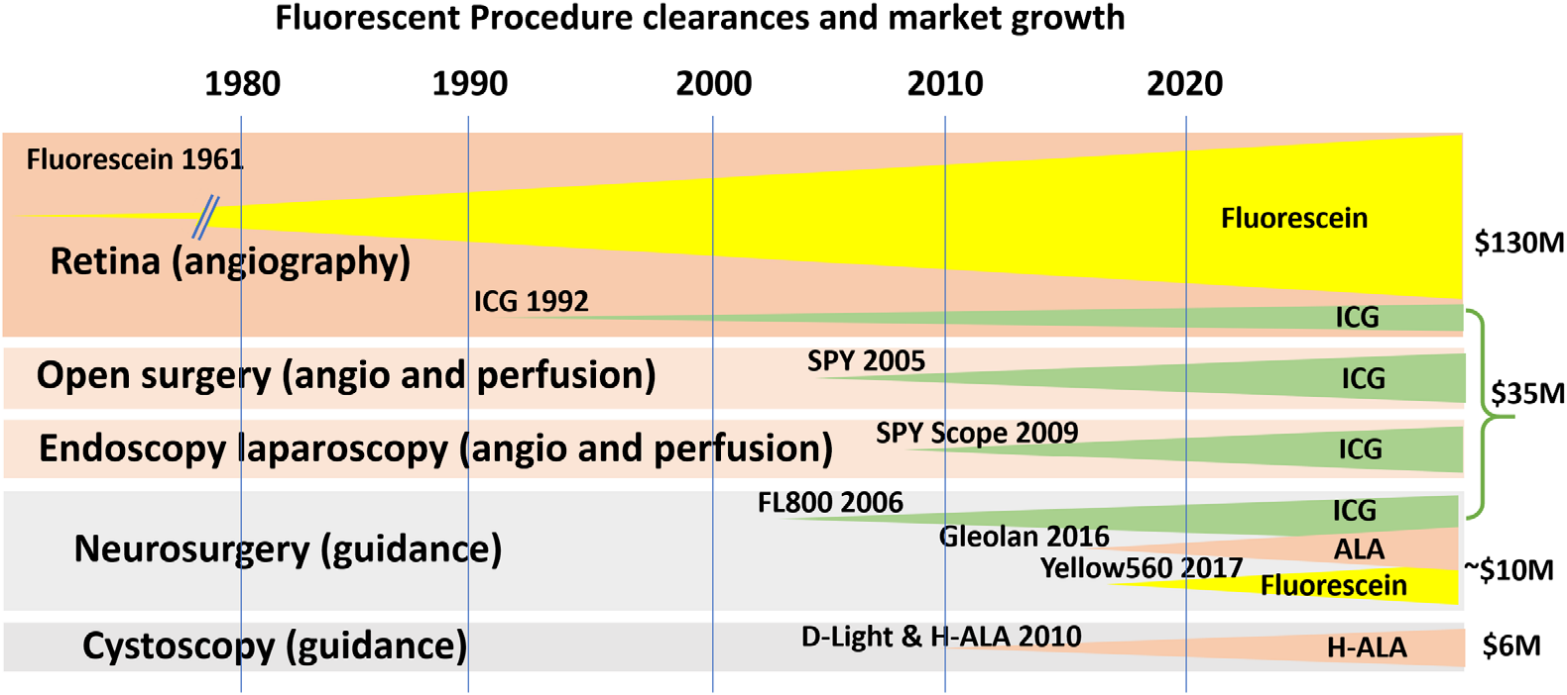
Fluorescent indications for surgical use all largely benefitted from the early indication approval of fluorescein-based retinal angiography, and the subsequent indication approval of NIR ICG-based angiography. The earliest approvals were in neurosurgery and tissue.

**Fig. 3:**
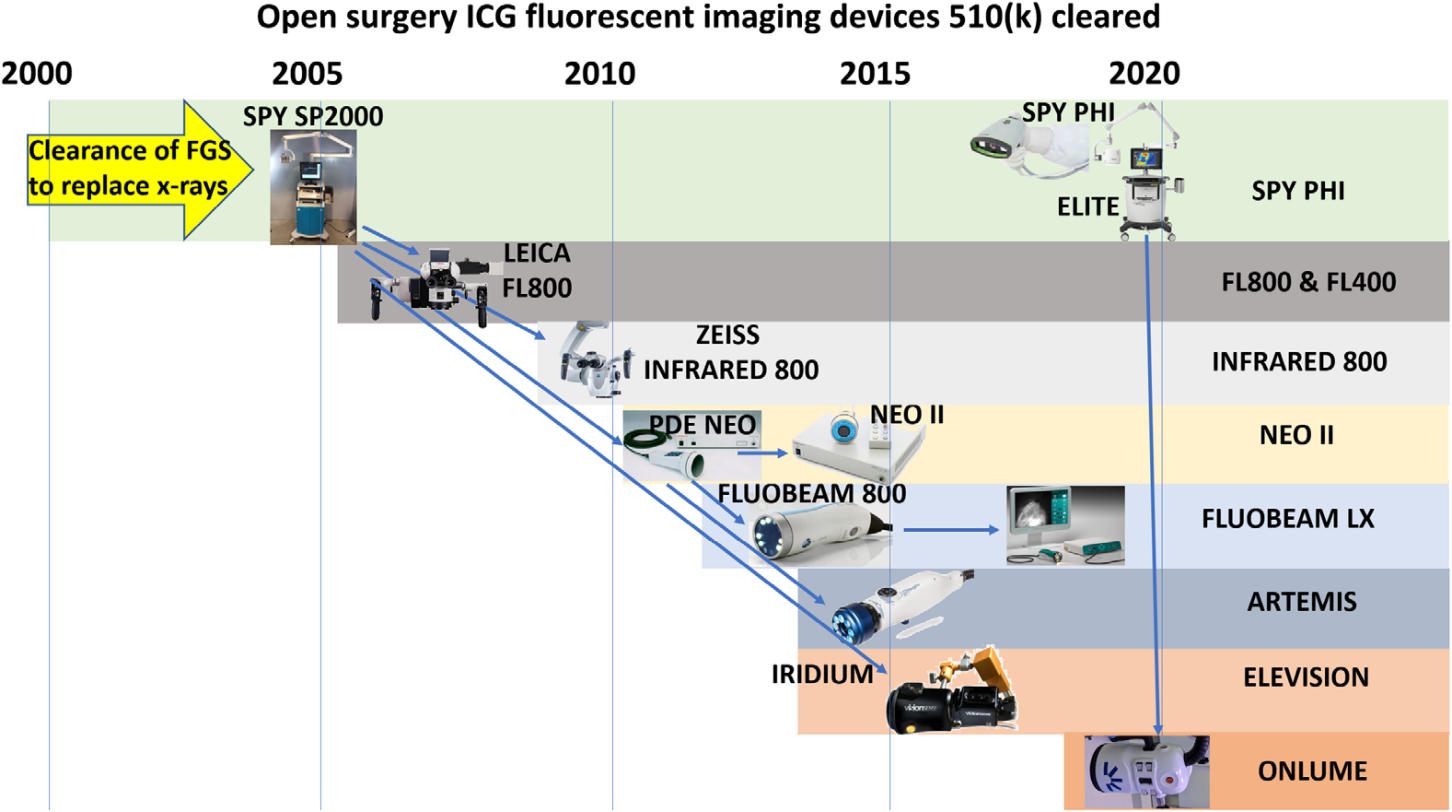
The pathways for modern FGS devices for open procedure use are illustrated, with many of the approvals pointing back to predicate devices that originate with the SPY SP2000 system. This first system was cleared for 510(k) equivalence based upon a predicate of an x-ray vascular imaging system, and retinal fluorescence angiography.

The translation of ICG fluorescence to other indications occurred through many investigator-led studies through the late 20th century, leading up to the first regulatory clearance application for use in surgical guidance by Novadaq with their 2005 clearance for the SPY SP2000 (see Fig. 2). The importance of this milestone is that this indication was approved with a new device, being based partially upon equivalence to x-ray vascular imaging of tissue, and partially upon fluorescence fundus imaging (see Fig. 3). This led to expanded use of ICG in a range of indications.

The newest class of agents approved for human use are ALA derivatives, which are the metabolic precursors to PpIX. Originally approved for PDT, it first found its role in surgical guidance first in the 2010 NDA from Photocure ASA as Cysview, in the form of hexaminolevulinate, to be instilled in the bladder for malignancy detection. This specific indication was coupled to the Karl Storz D-Light C Photodynamic Diagnostic system for blue-light cystoscopy. The next NDA for ALA was through the 2017 Gleolan NDA for neurosurgical resection guidance of glioma. This latter NDA was a milestone in that two imaging systems existed at the time of this NDA, and the approval was not tied to use of either of them.

The transition from ICG and fluorescein to ALA-based agents marks the transition from vascular/perfusion imaging to tissue-based metabolic probes. This brings with it a signal that is 100× lower than ICG and likely 1000× lower than Fluorescein. The need to have better background rejection is much higher, and the motivation to quantify the signal is higher, because it is not being used as a binary indication of flow, as ICG often is. Rather it is used as a probe of metabolism, which has large variations in production.

MB has never been approved as an FGS agent due to a range of reasons, including toxicity and low fluorescence yield. It is not clear that it will reach approval despite the large numbers of investigator-initiated human studies with it. Perhaps most importantly, MB is a generic imaging agent and used for a diagnostic purpose, as such most industry will be unlikely to accept the costs associated with advanced phase trials and so further development with it as an FGS agent is in question. It will likely remain an absorption-based agent for lymph node mapping, and the low emission yield may limit its use as it would have features such as ICG, but within the edge of the visible spectrum, which is less desirable technically because of room light contamination. However, its spectrum of absorption and emission can match that of ALA-PpIX, and so there may be synergy in the use of FGS systems designed for the latter, being applied to MB applications.

## 3 Surgical Procedure Indication Pathways

The origins of surgical guidance with fluorescence are intimately tied into the NDAs and device clearances, but the approval of a new indication can be accompanied by a new device, or the new indication can be approved for an existing device. Perhaps most interestingly, the existence of several cleared devices in laparoscopic and open surgery has led to growth in new indications with the same devices in recent years. In this section, indications are reviewed, which are sometimes tied into new devices, or sometimes just cleared based upon existing devices. These regulatory milestones about medical indication clearances and approvals were the keys to broadening use of FGS into different surgical specialties.

### 3.1 Open Surgery FGS Indications

The original clearance for the SPY SP2000 was for “intra-operative visual assessment of the coronary vasculature and bypass grafts during CABG surgery” (K042961, 2005). It was cleared as a combination product, whose primary mode of action was mediated by the device. This judgement by FDA allowed the SPY SP2000 to be cleared by 510(k) with the provision that the ICG be over labeled to include indications for use in angiography that specified dosage, route of administration, and period of imaging. The same device later became cleared for the more widely used indication of “visual assessment of blood flow as an adjunctive method for the evaluation of tissue perfusion, and related tissue transfer circulation in tissue and free flaps used in plastic, micro- and reconstructive surgical procedures.” (K063345, 2007). It was later cleared for surgeons to “visually assess blood flow and related tissue perfusion during organ transplant procedures” (K073130, 2008), and then for “visual assessment of arterial and venous blood flow and related tissue perfusion during GI surgical procedures” (K100371, 2010). With the development of the endoscopic systems, the Spy Scope (Pinpoint) was cleared (K091515, 2009), and its use was broadened (K150956, 2016) to a much wider indication band allowing “surgeons to perform minimally invasive surgery using standard endoscope visible light and visual assessment of vessels, blood flow and related tissue perfusion, and at least one of the major extra-hepatic bile ducts (cystic duct, common bile duct, or common hepatic duct), using near-infrared imaging.” Additionally, “fluorescence imaging of biliary ducts … with standard of care white light.” These broader indications allow a much wider range of use and facilitated larger growth in surgical use. In more recent clearances, it has been approved for interstitial administration of ICG and “intraoperative fluorescence imaging and visualization of the lymphatic system, including lymphatic vessels and lymph nodes” (K200737, 2020). This new route of administration for ICG required Novadaq to pursue a new NDA for its own ICG via the 505 (b) (2) pathway, for a brand of ICG that is specific to device clearances for lymphatic applications.^34^ There are other clearances for new systems and new packaged forms of ICG, but these clearances that broadened the use cases of ICG imaging have brought in other surgical specialties into the use of both the handheld open surgical systems and the endoscopic systems. More discussion of systems approvals is in the later section on New Device Clearances.

### 3.2 Neurosurgery FGS Indications

Neurosurgery FGS research existed as early as 1948 with fluorescein,^35^ although this was never approved for human use until 2017. The use of ICG for angiographic imaging was first approved in 2006 (K061871) by the Leica FL800, and later in 2010 (K100468) by Karl Zeiss INFRARED 800 and FLOW 800. While these were successful, the ability to see the surface vascular clarity of fluorescein was cleared in 2017 (K162991) in the Zeiss Yellow 560. Interestingly the predicate for this latter indication was the SPY SP2000 system, showing the lasting power of older established device predicates, even though not in the same indication.

Arguably the largest change in FGS occurred in 2017 with the NDA approval of Gleolan by NX Development Corporation as a metabolic indicator of tissue malignancy. What is most interesting about this NDA is that it was not linked to a device, although there were two devices used in early studies. The Zeiss BLUE 400 fluorescence system was able to be marketed directly as a Class I product, given that all surgical microscopes are viewed as Class I products. This was perhaps a final milestone in fluorescence guidance systems as low-risk devices. Part of the rationale for neurosurgical imaging devices being class I is that they are supplementary to the neurosurgeon directly viewing the fluorescence by their eye through the binoculars, and indeed all early imaging of ALA-PPIX in glioma neurosurgery was done by surgeon vision. Thus, the risk of the imaging system itself was viewed as low, given that it was considered supplementary to the neurosurgical procedure. However, by 2018, the Leica FL400 system was required to be cleared as a De Novo application (DEN180024), categorized as a class II device. This change in the regulatory pathway appears to have been related to the use of optical filters changing the information stream, and so the Leica FL400 system used the SPY SP2000 as an established predicate for vascular imaging with filtered fluorescent light imaging. These two devices, the Zeiss BLUE 400 and the Leica FL400, illustrate how complex and mixed the process to approve these devices can be, especially when they are approached as attachments or add on to existing systems, as is typically done in neurosurgery.

### 3.3 Endoscopic FGS Indications

The origins of endoscopic use with FGS has a longer history based around endogenous visible emission imaging. The first of these were approved in 1996 by Xillix in the Life-Lung bronchoscope as a PMA (P950042 S001),^36^^–^^38^ and later approved as the Onco-Life Endoscopic system (P950042 S003) in 2005,^39^^–^^41^ and subsequently re-branded (P950042 S003) by Novadaq in 2007.^42^ Modern use of exogenous agents with endoscopic systems started with the approval of the SPY Scope in 2009 (K091515) and rapidly expanded with robotic surgery use in 2010 with the Intuitive Surgical da Vinci Fluorescence Imaging Surgical System (K101077), and eventually the Firefly system in 2014 (K141077).^43^^–^^47^ Several other systems have been cleared as well, but the linkage to open surgery is less apparent, and so a detailed analysis of this surgical subspecialty is not the focus of this review. The market use for ICG has grown with these devices and will likely continue as procedures and adoption grows.

### 3.4 Bladder Indication

Cystoscopy with fluorescence has only one set of approvals that included both an NDA and a PMA for using hexaminolevulate HCl (CysView^®^) (NDA 022555) contrast that is instilled in the bladder, and visualization with the Karl Storz D-Light C system (P050027 S010). The development of this in Europe^48^^,^^49^ preceded the NDA approvals by the FDA in 2010 and the adoption of this methodology has been slow, due to the complexity of instillation and incubation time. Today though this indication appears promising, although issues around instillation into the bladder and integration into current practice is a topic of interest.^20^^,^^21^

## 4 New Device Clearances in Open Surgery—Predicates and New Pathways

Perhaps no other single clearance has been as important to open surgery as 2005 510(k) of the SPY Intraoperative Imaging System SP2000 to be used with ICG, developed by Novadaq Technologies Inc. (K042961). What makes this clearance most notable is that it had two predicate devices designated that were distinctly different than the applicant device, including the Philips Integra Series 2 Angiographic x-ray system (K984545) which was for diagnostic quality images during cardiac, vascular, neurovascular, and interventional applications. There was a second predicate device, the Heidelberg Retinal Angiographic System (K944261) which was for imaging the posterior segment of the eye and could be used with ICG. The applicants successfully argued that the SPY system could match combined functionality of the combination of these two predicates when imaging vascular perfusion. This kind of split predicate is less common in more recent device clearances. After this clearance, most 510(k) applications used this SPY device or equivalent devices to it, as their predicate (see Fig. 3). This pathway relies on the more obvious argument that the subsequent devices perform the same function of imaging tissue perfusion by fluorescence following intravenous administration of ICG. To achieve clearance, the SPY system had to show it complied with all IEC and UL requirements, and complete animal and human testing. Taken together this is an expensive and time-consuming series of studies. However, the lesson of this single clearance is that it is possible to make the argument that fluorescence imaging is substantially equivalent to x-ray imaging for the purposes of assessing vascular function.

Subsequent devices are shown in Fig. 3, where the cascade of clearances around the range of open surgery indications is clearly targeted. The five major companies that have advanced systems to the pre-market approval [Hamamatsu, Fluoptics, Quest Medical, Visionsense (now Medtronic), and OnLume], have each benefitted from the predicates available to them from the SPY Novadaq systems (now Stryker). These clearances are not surprising, and are rather an indication of a growing market that can likely support more than one system on the market. Discussion of the differences or critical considerations for these devices is outside the scope of this paper but was recently reviewed,^50^ however, it is worth commenting that light control (in/out/filtering/intensities) and software ergonomics are probably the key elements that will drive the success or failure of device.

## 5 Discussion

NDAs for fluorescence imaging agents have historically been driven by imaging agents that were not originally approved for use as fluorescence technology. ICG and fluorescein were originally approved as absorbing dyes. Similarly, MB and different forms of ALA were approved for their therapeutic value, not their fluorescence. It is fascinating to see how the lateral translation of these dyes into diagnostic use was facilitated by NDAs that were driven by different company efforts. In instances when imaging agent NDAs were approved and the imaging agent was available for immediate use with multiple devices (e.g., such as Gleolan with Zeiss and Leica microscopes), this was only possible because the devices used were historically classified as a surgical aid and Risk Class 1. More commonly, the barriers to approving a new imaging agent-device combination for surgical visualization are high, and this will likely continue to temper the rate of new market entries with the most successful entrants targeting only the most profitable procedures.

For FGS devices, PMA approvals are generally less common than 510(k) clearances, simply because of the investment required and the business risks involved in establishing a fundamentally new device and indication. The 510(k) pathway represents a lower regulatory burden and consequently also a lower risk proposition. Of course, the drawback of the 510(k) pathway is that the indications for use may be less compelling (as they are not life sustaining) and there is likely more competition in the market. The growth of newly cleared Risk Class II devices around the same set of indications has been what is occurring in open-field FGS systems, as shown in Fig. 3. The field is expanding and the indications for use with these devices are expanding, and so several companies are offering devices to serve the needs of specialty surgeons and their techniques.

One of the most striking approvals was the 510(k) clearance of the SPY SP2000, mostly because it was for an indication based upon predicate devices that were arguably quite different devices. The use of such a split predicate is less common in recent clearances, however, the SP2000 history demonstrates that when argued carefully, this approach has been strategically successful in gaining a market clearance which might otherwise have required a De Novo clearance or PMA approval. Both latter pathways are more complex in argument and carry a higher risk in approval. Perhaps most interestingly, although the SP2000 is no longer being manufactured, it remains the traceable root predicate in clearance of several subsequent devices from multiple companies. Clearly, this single approval is one of the major events in the history of FGS in open surgery.

As the field of FGS grows, these open surgery tools are now gaining a widespread adoption, and as surgeons recognize the capabilities of these tools, there is a lateral spread of the technology into other indications and/or other surgical specialties. These new indications for existing cleared FGS devices and imaging agents broaden the use driving FGS toward a standard of care and creating an environment that encourages further investment in the development and commercialization of this promising technology.
